# Detection of *Leishmania* DNA in Ticks and Fleas from Dogs and Domestic Animals in Endemic Algerian Provinces

**DOI:** 10.3390/microorganisms13102338

**Published:** 2025-10-10

**Authors:** Razika Benikhlef, Naouel Eddaikra, Assia Beneldjouzi, Maria Dekar, Lydia Hamrioui, Karima Brahmi, Souad Bencherifa, Denis Sereno

**Affiliations:** 1 Laboratory of Parasitological Eco-Epidemiology and Populations Genetics, Institut Pasteur d’Algérie, Route du Petit Staoueli, Dely Brahim, Algiers 16302, Algeria; 2 GoInsect: Infectiology and Entomology Research Group, 34000 Montpellier, France; 3 Faculté des Sciences de la Nature et de la vie, Université de M’hamed Bouguerra, Boumerdes 35000, Algeria; 4 Laboratoire d’Écologie et de Biologie des Écosystèmes Terrestres, Université Mouloud Mammeri, Tizi-Ouzou 15000, Algeria; 5 UMR177 Intertryp, Institut de Recherche pour le Développement, CIRAD, University of Montpellier, 34000 Montpellier, France

**Keywords:** *Leishmania*, ectoparasites, ticks, fleas, Algeria, reservoir hosts, molecular detection

## Abstract

Background: *Leishmaniasis* is a zoonotic vector-borne disease and a significant global public health concern worldwide and in Algeria. In this study, we investigated the potential role of ticks and fleas as carriers of *Leishmania* in endemic regions of Algeria. Methods: Adult ectoparasites were collected from reservoir dogs and cohabiting animals across three provinces: Tizi-Ouzou (northeast), M’Sila (southeast), and Tébessa (extreme east). A subset of 247 ectoparasites was randomly selected for *Leishmania* DNA screening using ITS1-PCR. Results: Morphological identification revealed two tick species, *Rhipicephalus turanicus* (378 specimens) and *Rhipicephalus sanguineus s.l* (127 specimens), and one flea species, *Ctenocephalides felis* (94 specimens). Dogs were the most heavily infested hosts (74.12%), followed by sheep (9.51%) and cats (9.34%). *Leishmania* DNA was detected in 36.43% (90/247) of the tested specimens, with higher positivity in ticks (41.32%) compared to fleas (17.64%). Infection rates varied by host species, with dogs harboring the majority of positive ectoparasites (62/90), primarily *R. sanguineus s.l* (19/30) and *R. turanicus* (40/115). *Leishmania* DNA was also detected in ectoparasites collected from cats and sheep, whereas goats and rabbits were free from *Leishmania* DNA. Conclusions: This investigation highlights the high detection rate of *Leishmania* DNA in ticks and fleas from animals in Algerian endemic regions, indicating exposure to infected hosts. Together with previous reports, these findings support the view that ticks and fleas may act as incidental hosts or mechanical carriers of the parasite. However, their role in parasite transmission remains unconfirmed and warrant further investigation, particularly through studies assessing vector competence. These results emphasize the need for additional research to clarify the contribution of these ectoparasites to *Leishmania* transmission and multi-host dynamics.

## 1. Introduction

Leishmaniasis is a neglected zoonotic vector-borne disease and a major global public-health challenge; sand flies remain the only proven biological vectors [1,2]. Infection occurs when humans, sandfly vectors, and reservoir hosts coexist within shared ecological niches [3,4]. Clinical manifestations vary from self-limiting cutaneous lesions to destructive mucosal tissue damage and potentially fatal visceral dissemination in untreated cases [5].

The World Health Organization (WHO) estimates that approximately 350 million people are at risk of Leishmaniasis, with nearly 12 million reported infections across four continents [2,6]. In 2020, Algeria ranked as the second-most affected country globally for cutaneous leishmaniasis (CL), following Afghanistan, among the 200 nations reporting data to the WHO. Over the past three decades, more than 250,000 cases have been documented across all 48 provinces in Algeria [7,8,9]. The country’s ecological diversity, spanning coastal zones, wetlands, subhumid regions, semi-arid and arid areas, deserts, oases, and densely forested mountains supports a wide variety of *Leishmania* reservoir hosts and vector species [10,11,12].

The hypothesis that sandflies serve as vectors for *Leishmania* was initially proposed by Sergent brothers, and this proposition gained further support in 1907, when Patton identified flagellated forms of *Leishmania* within the gastrointestinal tract of sandflies [13,14,15,16]. The vectorial role of sandflies was later experimentally validated by the Adler and Sergent brothers at the Pasteur Institute of Algeria [15,16]. Simultaneously, Patton and Wenyon considered alternative transmission pathways, hypothesizing that ticks, bed bugs, and fleas may also act as a bridge vector [17,18]. Subsequent research has focused on the susceptibility of these arthropods to the parasite [19,20]. Among the 25 sandfly species recorded in Algeria, five are recognized as major vectors of *Leishmania*. *Phlebotomus perniciosus*, *P. perfiliewi*, and *P. longicuspis* transmit *L. infantum*, the causative agent of visceral leishmaniasis (VL), sporadic cutaneous leishmaniasis (SCL), and canine leishmaniasis (CanL); *Phlebotomus papatasi* transmits *L. major*, responsible for zoonotic cutaneous leishmaniasis (ZCL); *P. sergenti* is implicated in the transmission of *L. tropica*, the etiological agent of the anthroponotic cutaneous leishmaniasis (ACL) [9]. In Algerian endemic foci, infection rates in sandflies are usually around 5% [21].

*Leishmania infantum*, the causative agent of visceral leishmaniasis (VL), some form of CL, and canine leishmaniasis (CanL) [9,22], persists in peridomestic environments through its primary reservoir, domestic dogs. These animals pose significant zoonotic risks because of their close proximity to humans [23]. Both viable parasites and *Leishmania* DNA have been detected in ticks and fleas that infect dogs and cats [24,25,26,27,28,29,30]. Ticks and fleas are characterized by their widespread distribution, high reproductive rates, and dense host-dependent populations. They are recognized vectors of multiple vertebrate pathogens. In addition, molecular studies have identified *Leishmania* DNA in *Rhipicephalus sanguineus s.l* and *Ixodes ricinus* ticks collected from dogs and cats in Italy [27,31,32,33,34] and in *Ctenocephalides felis* fleas from Brazilian dogs [23]. Nevertheless, the role of bridge vectors, notably the brown dog ticks *R. sanguineus s.l*, *R. turanicus*, and the cat flea *C. felis* in *Leishmania* transmission remains largely understudied [35]. Accordingly, detecting *Leishmania* DNA in ticks and fleas can serve as a pragmatic proxy for parasite circulation in peridomestic animals, even if these ectoparasites are not proven vectors

Although the role of ticks and fleas in the epidemiology of leishmaniasis remains debated, documenting their contact with *leishmania*, is important to delineate the extent to which they may act as incidental carriers or hosts. Continued surveillance across endemic regions is therefore important to better characterize parasite–ectoparasite associations. Positioning ectoparasite DNA screening within a One Health framework strengthens surveillance by explicitly linking veterinary reservoirs and human risk. In this context, we aimed to detect *Leishmania* spp. DNA in ticks and fleas collected from animals in Algerian regions endemic for cutaneous, visceral, and canine leishmaniasis, thereby contributing additional data to the body of evidence on their potential involvement in parasite circulation, and providing regional epidemiological insights, thereby contributing evidence on their potential involvement in parasite circulation and delivering region-specific epidemiological insights from Algeria.

## 2. Materials and Methods

### 2.1. Study Area

**Figure 1:**
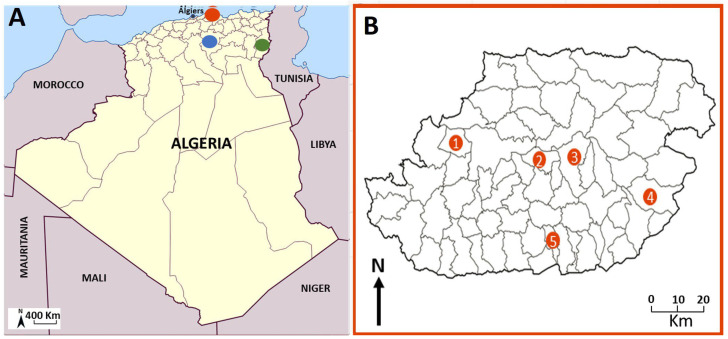
(**A**) Geographical distribution of the three sampling regions: (•) Tizi Ouzou, (•) Msila (•) Tébessa (**B**). Location of the five sampling stations within Tizi-Ouzou Province ((1) Draâ Ben Khedda, (2) Tizi Rached, (3) Mekla, (4) Bouzgeune, (5) Ouacif).

This study was conducted across three provinces (wilayas) in Algeria: Tizi-Ouzou (northeast), M’Sila (southeast), and Tébessa (extreme east, adjacent to Tunisia) (Figure 1). Tizi-Ouzou, situated 100 km from Algiers in the Kabylie region, is a known area for VL and CanL caused by *L. infantum*. The region is characterized by a humid to subhumid climate and features mountainous terrain within the Djurdjura Massif. M’Sila and Tébessa, located 240 km and 612 km east of Algiers, respectively, are endemic areas for ZCL [8,36]. These regions are situated within the semi-arid to arid high plateaus of Algeria (Figure 1A,B).

### 2.2. Sampling Collection

**Figure 2:**
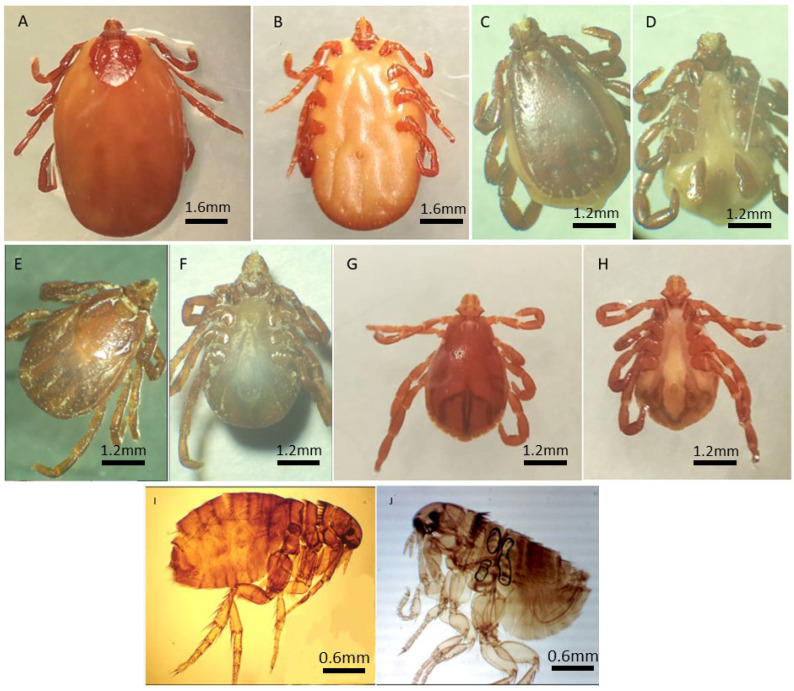
Morphological identification of adult’s ticks and fleas. *R. turanicus* ♀ ((**A**) dorsal view, (**B**) ventral view); *R. turanicus* ♂ ((**C**) dorsal view, (**D**) ventral view); *R. sanguineus s.l* ♀ ((**E**) dorsal view, (**F**) ventral view); *R. sanguineus s.l* ♂ ((**G**) dorsal view, (**H**) ventral view); *C. felis* ♀ (**I**) and *C. felis* ♂ (**J**) Light stereomicroscope View (Magnification ×20).

Adult ectoparasites were collected from dogs showing no evident symptoms of leishmania infection, as well as from their cohabiting animals, including cats, sheep, goats, and rabbits, between December 2023 and June 2024. The feeding status of the collected ectoparasites was not recorded at the time of collection. In the Tizi-Ouzou region, ticks and fleas were collected from five districts (daïras), namely: Draâ Ben Khedda, Ouacif, Sidi Rached, Bouzguene, and Mekla, specifically between March and May 2024 (Figure 1, Table 1). These sampling sites were chosen based on documented occurrences of autochthonous VL and CanL [37,38]. In Tébessa and M’Sila, a limited number of specimens were collected in December 2023 and May 2024, respectively, from two specific sites: Messloula (El Aouinet District, northern Tébessa) and M’Sila city (provincial capital) (Figure 2). The ectoparasites were extracted using fine forceps for ticks and brushes for fleas during comprehensive examination of sedated animals. The specimens were preserved in 70% ethanol for further analysis.

### 2.3. Morphological Identification of Ticks and Fleas

Morphological assessments were conducted at the Laboratory of Parasitic Eco-Epidemiology and Population Genetics, Pasteur Institute of Algiers. The examinations employed both a stereomicroscope and light microscope, with magnifications ranging from 10× to 40× for the stereomicroscope and 100× for the light microscope. Taxonomic identification adhered to established keys for ticks [39,40] and fleas [41,42].

#### 2.3.1. Tick Identification

Ticks were cleaned with distilled water and examined for diagnostic features such as: rostrum size/shape, anal groove position relative to the anus, presence/absence of festoons and eyes, ventral plate morphology, and capitulum base structure [39]. Species identification relies on adanal plate characteristics in males and scutum shape/genital aperture morphology in females [40,43]. Additionally, species identification was determined based on supplementary characteristics, including size, presence of festoons, structure of coxae, and number and shape of adanal plates in males, as well as the shape of the scutum and genital orifice in females [40]. Specimens belonging to the brown dog tick were identified morphologically as *Rhipicephalus sanguineus sensu lato* (*s.l*.). This taxon is currently recognized as a species complex comprising at least two cryptic species: the temperate lineage (*R. sanguineus* s.s) and the tropical lineage (*R. linnaei*) [44]. Because morphological criteria do not allow reliable distinction, we report our material under *R. sanguineus s.l.*

#### 2.3.2. Flea Identification

Key identification criteria included body shape, presence of ctenidia, leg structure/position, abdominal size, and head/thorax morphology [41]. The fleas were clarified in 10 to 20% KOH solution for a duration 12 to 24 h, followed by rinsing with distilled water. After dehydration using a graded ethanol series, they were mounted on slides using Canada balsam. Genus’s identification was conducted using identification keys from [41] which are predicated on the presence or absence of ctenidia (bristle combs) on the head and/or thorax, morphology of the antennae, and length of the hind legs. For instance, the genus *Ctenocephalides* is characterized by well-developed ctenidia, whereas Pulex lacks them. Upon determination of the genus, species identification was based on specific morphological characteristics, such as the shape of the abdominal segments and arrangement of the bristles, as outlined by Beaucournu and Launay [42].

### 2.4. Leishmania Molecular Detection

DNA was extracted individually from ethanol-preserved specimens using a modified cetyltrimethylammonium bromide (CTAB) protocol [45]. Adult ticks and fleas were dissected under a stereomicroscope, and internal tissues were homogenized in CTAB buffer with β-mercaptoethanol. After overnight incubation at 65 °C, DNA was precipitated with phenol-chloroform-isoamyl alcohol, 99% propanol, and 70% ethanol, eluted in 50 µL Tris-EDTA buffer and stored at −20 °C.

A subset comprising 40% of ectoparasites, categorized by collection site, host, and species, was screened for *Leishmania* via ITS1-PCR. Each reaction mixture (25 µL) included 2.5 µL of DNA, 4.0 mM MgCl_2_, 200 mM dNTPs, 500 nM for each primers (LITSR: 5′-CTGGATCATTTTCCGATG-3′; L5.8S: 5′-TGATACCACTTATCGCACTTA-3′), and 2 units of Taq polymerase. The thermal cycling protocol consisted of an initial denaturation at 94 °C for 4 min, followed by 35 cycles at 95 °C for 40 s, 53 °C for 30 s, and 72 °C for 60 s, and a final extension phase at 72 °C for 6 min [46]. Positive and negative controls were established using *Leishmania* reference strain DNA and nuclease-free water, respectively. The amplified products were analyzed by electrophoresis on 1% agarose gels, visualized under UV illumination, and compared to reference bands measuring 300–350 bp.

### 2.5. Statistical Analysis

#### 2.5.1. Multivariable Analysis of Factors Associated with Leishmania DNA Detection in Ectoparasites

A one-way ANOVA was performed using GraphPad Prism 8 to evaluate differences in ectoparasite abundance among the five Tizi-Ouzou sites, as well as for *Leishmania* detection. The relationship between ectoparasite sex and *Leishmania* infection was ascertained using the chi-square test. Both analyses involved the evaluation of *p*-values to assess statistical significance, with a threshold set at *p* < 0.05.

#### 2.5.2. Logistic Regression Model

Logistic regression analysis was conducted to assess whether ectoparasite species and host animal type were significant predictors of *Leishmania* DNA detection. Data were extracted from Table 1, which reported the number of positive and tested ectoparasites across combinations of three ectoparasite species (*R. sanguineus s.l*, *R. turanicus*, and *C. felis*) and three host animals (dog, cat, and sheep). These counts were expanded into individual binary observations, where each ectoparasite was treated as a data point coded as 1 (positive) or 0 (negative) for *Leishmania* DNA. Ectoparasite species and host type were included as categorical independent variables, with *C. felis* and cat serving as the reference categories. A binomial logistic regression model was fitted using the Statsmodels package in Python (v0.13.5), and model outputs included coefficients, standard errors, z-scores, *p*-values, and 95% confidence intervals.

### 2.6. Ethical Considerations

The study protocol was approved by the Ethics Committee of the Pasteur Institute of Algeria (Helsinki Declaration, 1964). Animal procedures complied with the guidelines of the Algiers Veterinary Inspection Department (Veterinary Health Declaration No. 2505/IVWA/2019, April 2020) and the Algerian Association for Animal Experimentation Sciences (AAAES). Informed consent was obtained from all the animal owners.

## 3. Results

### 3.1. Morphological Identification of Ticks and Fleas

Specimens were identified using standard taxonomic keys as described in Section 2 (Figure 2). A total of 599 ectoparasites were collected from domestic animals across the seven localities in three Algerian provinces: Tizi-Ouzou (northeast), M’Sila (southeast), and Tébessa (east). Morphological identification revealed three species: two hard ticks, *R. turanicus* and *R. sanguineus s.l.,* and one flea, *C. felis*.

#### 3.1.1. Geographic Distribution

**Figure 3:**
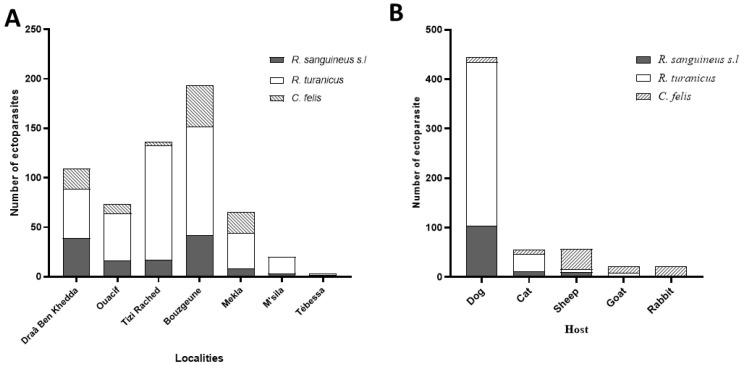
Distribution of ectoparasites across various capture locations (**A**) and among different host species (**B**).

Ectoparasite abundance varied among localities. The majority of specimens (n = 389; 64.53%) were collected in the Tizi-Ouzou province, with the highest counts recorded in Bouzguene (n = 193; 33.5%), Tizi Rached (n = 136; 23.61%), and Draâ Ben Khedda (n = 109; 18.92%). Lower numbers were obtained from Ouacif (n = 73) and Mekla (n = 65), despite their similarly high-altitude settings. This discrepancy may be partially attributed to differences in acaricide use and dog management practices [47,48]. Nevertheless, statistical analysis did not reveal significant differences in ectoparasite abundance among the five Tizi-Ouzou sites (one-way ANOVA, *p* > 0.05; Figure 3A). In comparison, substantially fewer ectoparasites were collected in M’Sila (n = 20) and Tébessa (n = 3). No flea specimens were recorded from these localities. *R. turanicus* was dominant in M’Sila (85% of local specimens). The low yields in these provinces may reflect seasonal timing of collection or the impact of vector control campaigns, especially in M’Sila, a region known for recent leishmaniasis activity [8].

#### 3.1.2. Host Distribution

Ectoparasites were collected from a variety of domestic animals, with dogs accounting for the majority of infestations (74.12%; n = 444). Other host contributions were as follows: sheep (n = 89), cats (n = 56), and goats and rabbits (n = 21). Dogs are recognized as the primary domestic reservoir of *L. infantum* in Algeria [49,50].

#### 3.1.3. Species Composition and Prevalence

Among the 599 ectoparasites identified: *R. turanicus* was the most prevalent (n = 378; 217♀, 161♂), followed by *R. sanguineus s.l.* (n = 127; 62♀, 65♂), *C. felis* was less common (n = 94; 80♀, 14♂). Dogs were infested predominantly with *R*. *turanicus* (n = 332; 188♀, 144♂), followed by *R. sanguineus s.l* (n = 103; 44♀, 59♂), and a small number of *C. felis* (n = 9; 7♀, 2♂). Sheep (n = 89) were mostly infested with *C. felis* (n = 41; 34♀, 7♂), along with *R. sanguineus s.l* (n = 10; 7♀, 3♂) and *R. turanicus* (n = 6; all♀). Cats (n = 56) were primarily infested with *R. turanicus* (n = 34; 17♀, 17♂), *R. sanguineus s.l* (n = 12; 9♀, 3♂), and *C. felis* (n = 10; all♀). Goats and rabbits (n = 21): Goats hosted *R. turanicus* (n = 6; all♀), *R. sanguineus s.l* (n = 2; all♀), and *C. felis* (n = 13; 9♀, 4♂); rabbits were parasitized exclusively with *C. felis* (n = 21; 20♀, 1♂). The observed female-biased sex ratio across all species may reflect differences in host-seeking behavior or the timing of sampling, although these trends were not statistically assessed in this study. While ticks represented the majority of total ectoparasites, *C. felis* exhibited notably broad host diversity, being recovered from five different species. The highest infestation rates were seen in sheep (43.61% of their ectoparasites), followed by goats (13.82%), Cats (10.63%), Dogs (9.57%), and Rabbits (100%). These patterns are consistent with previous reports from Algeria indicating widespread parasitism of *C. felis* across mammalian hosts, especially felines and small ruminants [51,52]. While *C. felis* was not found in M’Sila or Tébessa, this may be influenced by regional climate conditions or control measures in endemic zones.

### 3.2. Detection of Leishmania DNA in Ectoparasites

**Figure 4:**
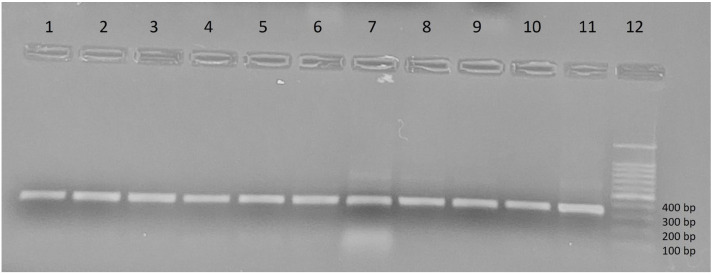
Amplification profile of the *Leishmania* ITS1 gene visualized on a 1% agarose gel. Lanes 1–4: samples of *R. sanguineus s.l*, lanes 5–8: samples of R. *turanicus*, lanes 9–10: samples of *C. felis*, lane 11: positive control (reference strain LEM75/*L. infantum* MON-1), lane 12: DNA Ladder.

Out of the 599 collected ectoparasites, a subset of 247 specimens (41.23%) was randomly selected for molecular screening of *Leishmania* DNA. These include representatives from all three study provinces and were distributed as follows: Tizi-Ouzou (224: 102 from Tizi Rached, 60 from Bouzguene, 32 from Ouacif, 19 from Draâ Ben Khedda, 11 from Mekla), M’Sila (20), and Tébessa (3) (Table 1). Polymerase chain reaction amplification of the ITS1 region detected *Leishmania* DNA in 90 of the 247 tested specimen (36.43%) (Table 1, Figure 4). This result indicates a substantial level of exposure among ectoparasites, with infection rates varying across arthropod species, host animals, and sampling location.

#### 3.2.1. Leishmania DNA Prevalence by Ectoparasite Species and Host Animal

Of the three ectoparasite species screened, ticks exhibited higher DNA positivity compared to fleas: *R. sanguineus s.l* had the highest prevalence at 46.34% (19/41; 12♂, 7♀), and *R. turanicus* followed with 40.0% (62/155; 24♂, 38♀). *C. felis* exhibited the lowest positivity rate at 17.64% (9/51), with infections limited to females. Overall, ticks were significantly more likely to harbor *Leishmania* DNA than fleas, with detection in 41.32% of ticks compared to 17.64 in fleas (9/51).

When stratified by host species, dogs accounted for the highest number of positive ectoparasites, representing for 62 of the 90 positive cases (68.8%), primarily involving *R. sanguineus s.l* (19/30) and *R. turanicus* (40/115). Cats contributed 23 positive ectoparasites (25.55%), mainly *R. turanicus s.l* and *C. felis*. Sheep showed 5 positive ectoparasites (5.55%), including 3 *C. felis* and 2 *R. turanicus*. No positive samples were detected in ectoparasites from goats or rabbits.

#### 3.2.2. Geographic Variation in Leishmania DNA Detection Rate

Results are presented in Table 2. Among the 224 specimens screened from Tizi-Ouzou, 68 tested positive for *Leishmania* DNA (30.35%). Infection prevalence varied across localities. Drâa Ben Kheda recorded the highest infection rate (68.42%, 13/19), including *R. turanicus* (6/10), *R. sanguineus s.l* (5/7), and *C. felis* (2/2). Ouacif showed 40.62% positivity (13/32), with DNA presence in both tick species. Tizi Rached had DNA detection rate of 37.25% (38/102), including high DNA detection levels in *R. turanicus* (30/91) and 100% in *C. felis* (3/3). Bouzguene presented a lower detection ration (6.66%; 4/60), with all positive cases in *C. felis*. Mekla yielded no positive samples among 11 *C. felis* specimens.

Notably, the province of M’Sila and Tébessa, although represented by fewer samples, exhibited remarkably high infection rates. In M’Sila, *Leishmania* DNA was detected in 100% of the 17 *R. turanicus* and 3 *R. sanguineus s.l* tested. In Tébessa, 2 out of 3 ticks tested positive (66.66%), one each from R. *turanicus* and *R. sanguineus s.l*.

While ticks are not established biological vectors of human or animal *Leishmania* pathogens, the presence of *Leishmania* DNA in these ticks suggests exposure to infected hosts. This finding indicates that these regions may have a higher prevalence of *Leishmania* infection among domestic animals, particularly dogs, which are known reservoirs of the parasite.

#### 3.2.3. Host Specific Leishmania DNA Detection Patterns

**Figure 5:**
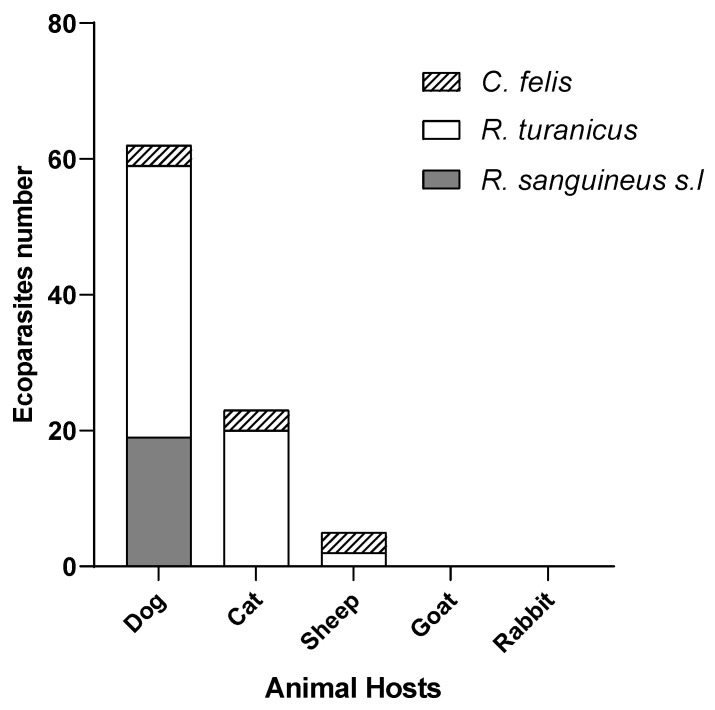
Hosts distribution of ectoparasites harboring *Leishmania* DNA.

Further analysis revealed notable host vector interactions (Table 3, Figure 5). Dogs were consistently the main source of infected ticks across most regions, especially in Draâ Ben Khedda, and Tébessa. In contrast, in Tizi Rached and Bouzquene, cats contributed a higher proportion of positive ectoparasites, particularly *R. turanicus* and *C. felis*. In Tizi Rached, 58.82% (20/34) of R. *turanicus* ticks collected from cats carried *Leishmania* DNA, and in Bouzguene, 30% (3/10) of *C. felis* from cats were positive. In sheep, *Leishmania* DNA was mostly detected in *C. felis* (3/14; 21.42%) and *R. turanicus* (2/6; 33.33%), while no *R. sanguineus s.l* ticks from sheep were positive.

#### 3.2.4. Influence of Arthropod Sex on Leishmania DNA Detection Rate

The sex ratio of positive ectoparasites was female-biased at 1.5. However, statistical analysis using the chi-square test showed no significant association between ectoparasite sex and *Leishmania* infection status (*p* > 0.05), suggesting that both sexes are equally likely to acquire the pathogen (Table 4).

#### 3.2.5. Logistic Regression Analysis

A logistic regression model was used to evaluate the association between ectoparasite species, host animal type, and the likelihood of detecting *Leishmania* DNA. Using *C. felis* and cats as reference categories, the model estimated the odds of positivity for other combinations based on 247 ectoparasites represented in expanded binary form (Table 5). Although *Rhipicephalus sanguineus* showed a slightly increased odds ratio (OR = 1.16; *p* = 0.804) and *R. turanicus* a decreased odds (OR = 0.89; *p* = 0.819), these associations were not statistically significant. Similarly, ectoparasites from dogs (OR = 0.95; *p* = 0.882) and sheep (OR = 0.37; *p* = 0.113) did not show significant differences in infection odds when compared to cats. The confidence intervals for all predictors included 1.0, indicating a lack of statistical association. Overall, the model did not identify any significant predictors of *Leishmania* DNA positivity, likely due to the limited sample size, particularly in the flea and non-dog categories. These findings highlight the need for larger, more balanced datasets to better understand host and vector factors influencing parasite presence in ectoparasite populations.

## 4. Discussion

Ticks and fleas are obligate hematophagous ectoparasites that infest a wide range of vertebrates and transmit numerous bacterial, viral, and protozoan pathogens of medical and veterinary importance. Ticks, classified under the order Ixodida, comprise over 900 species across three families: Ixodidae (hard ticks), Argasidae, (soft ticks); and Nuttalliellidae [39,53]. Fleas, belonging to the order *Siphonaptera*, are closely associated with their hosts and are globally distributed, with more than 2574 species and subspecies recorded since 1979 [54,55]. Ticks and fleas are not proven biological vectors of *Leishmania*; nonetheless, DNA detection in these ectoparasites is frequent across settings, including Algeria (this study). In this role, ticks and fleas function *as sentinel organisms*, signalling community-level parasite burden in dogs and other hosts.

In Algeria, *R. sanguineus s.l., R. turanicus*, and *C. felis* are the most epidemiologically relevant ectoparasites, due to their broad host range and adaptability to diverse ecological conditions [56,57,58,59]. The dominance of *R. turanicus*, especially on dogs and cats, contrast with historical findings where *R. sanguineus* and *R. bursa* were more prevalent [47,48]. This shift may reflect changes in host community structure and regional management practices, such as the use of acaricides, alongside broader ecological drivers like climatic variation and habitat modification. The adaptability of *R. turanicus* to both humid and semi-arid climates align with its seasonal bimodality and supports its role in diverse transmission settings [60,61,62]. Our data support body of evidence suggesting that these species, particularly *R. turanicus*, may contribute to *Leishmania* maintenance in Algerian Leishmaniasis-endemic zones, through contact with infected hosts. Similarly, the detection of *R. sanguineus s.l* across multiple host types, including wildlife, reinforces its continued relevance as a bridge vector in peri-domestic contexts [47,59,63,64,65].

The presence of *C. felis* on both carnivores and small ruminants confims its broader host spectrum, consistent with reports from overlapping domestic-animal communities [51]. Although less abundant than ticks, 17.64% of female fleas were positive for *Leishmania* DNA, in line with previous studies from Brazil and Italy [28,66]. While vertical or transstadial transmission has not been demonstrated the higher detection in females may reflect behavioral differences in host interaction, increasing exposure to infected blood meals.

The observed geographic and host-related heterogeneity helps pinpoint **micro-foci** of elevated transmission risk within provinces and even between nearby districts. Geographic and host-related variation in ectoparasite distribution underscores the complexity of vector ecology in Algeria. For example, sites such as Mekla and Ouacif, showed lower ectoparasite prevalence, which could be influenced by differences in veterinary management practices or environmental conditions [47,48]. Conversely, the similar species composition within Tizi-Ouzou sites suggests the presence of a broadly suitable ecological niche, shaped by microclimatic stability and steady host availability.

Although *Leishmania* DNA was detected in *R. turanicus*, *R. sanguineus s.l*, and *C. felis*, this alone does not confirm vector competence. Sandflies remain the only proven vectors, with infection rate around 5% [21]. By contrast, we observed markedly higher detection rates in ectoparasites 36.43% (41.32% in ticks and 17.64% in fleas). Our findings do not warrant recognition of ticks and fleas as vectors but do raise important questions about their potential role in parasite circulation, especially in contexts of sporadic sandfly activity or ineffective vector control. Passive carriage, mechanical transfer, or contamination from infected blood remains a plausible explanation. Nevertheless, the increasing detection of *Leishmania* in non-sandfly arthropods demands further exploration of alternative transmission routes in regions with sporadic sandfly activity or failed vector control [30,32]. Because it is low-cost and host-centered, ectoparasite monitoring can augment routine surveillance—particularly valuable in resource-limited settings or during periods of low sandfly activity.

Dogs, the primary reservoir hosts, accounted for the majority of positives, followed by cats, which may represent an underappreciated reservoir. The increasing documentation of feline leishmaniasis across the Mediterranean Basin reinforces this concern [67,68,69,70].

While descriptive data suggested difference in positivity rates between *R. sanguineus s.l* compared to *R. turanicus*, multivariate logistic regression revealed no statistically significant associations between ectoparasites species or host animal and *Leishmania* DNA detection. The lack of significant predictors is likely attributable to small and uneven sample sizes, particularly *C. felis* and non-canine hosts, which limited statistical power. Within the available dataset, no single ectoparasite–host combination independently predicted infection risk when adjusted for other variables. These limitations highlight the need for larger, more balanced datasets through systematic sampling across host species, ectoparasite taxa, and ecological zones. Such efforts are essential to identify the ecological and epidemiological drivers of *Leishmania* detection and to strengthen future models that incorporate parasite load, host infection status, and environmental variables.

PCR remains a highly sensitive tool for detecting parasite DNA, yet it does not establish viability or infectivity. It is a tool of interest for initial screening and evaluating the distribution of parasite DNA within ectoparasite populations. DNA traces may simply reflect environmental exposure or previous interactions. Nonetheless, PCR provides a valuable first step in mapping parasite distribution within ectoparasite populations. Complementary approaches such as sequencing, RFLP, or quantitative PCR (qPCR) would improve species confirmation and differentiation of sympatric *Leishmania* strains, particularly *L. infantum*.

A limitation of our study is the absence of data on feeding status. Distinguishing between engorged and questing ectoparasites would provide important insights, as persistence of *Leishmania* DNA after molting is more epidemiologically meaningful than detection in recently engorged specimens.

To clarify the role of ticks and fleas in the epidemiology of leishmaniasis, future studies should combine molecular detection with parasite culture, viability assays, and experimental vector competence trials. Investigating parasite survival through molting, transmission efficiency, and ectoparasite immune interactions will be essential. Integrating microbiome analyses (e.g., midgut microbial communities) may also help reveal cofactors influencing parasite persistence in non-traditional vectors [71]. Finally, longitudinal surveys will be crucial to assess seasonal patterns and the persistence of *Leishmania* DNA in ectoparasite populations, thereby providing a more dynamic understanding of parasite ecology.

## 5. Conclusions

Our findings contribute to the growing body of evidence that *Leishmania* DNA can be frequently detected in ticks and fleas. This study provides the molecular confirmation of *Leishmania* DNA in Algerian ectoparasites, indicating their exposure to infected hosts. Together with previous reports, these results support the view that ticks and fleas may act as incidental hosts or mechanical carriers of the parasite [18]. Nevertheless, our data do not confirm any role in parasite transmission. Future work should prioritize assessing parasite viability (culture/qPCR), persistence through molting, and experimental vector competence to determine whether any biological transmission is possible.

## Figures and Tables

**Table 1:** Sources and molecular results of *Leishmania* spp. detected in adult ticks and fleas collected from various hosts in northeastern (Tizi-Ouzou), southeastern (M’sila), and extreme eastern (Tébessa) Algeria between December 2023 and May 2024.

Samples Sites and GPS		Hosts	Number of Ticks or Fleas per Animal Host	Ticks or Fleas Species	Sex of Ticks/Fleas		*Leishmania* DNA Detection (n)	
					Female	Male	Negative	Positive
Draâ Ben Khedda	36°44′06″ N, 3°57′20″ E	Dog	5	*R. sanguineus s.l*	5	0	0	5♀
			4	*R. turanicus*	0	4	0	4♂
		Dog	24	*R. sanguineus s.l*	14	10	_/	_/
			40	*R. turanicus*	24	16	/_	_/
		Dog	6	*C. felis*	4	2	_/	_/
		Sheep	2	*R. sanguineus s.l*	1	1	2	0
			6	*R. turanicus*	6	0	4	2♀
		Sheep	8	*R. sanguineus s.l*	6	2	/_	/_
		Sheep	2	*C. felis*	2	0	0	2♀
		Sheep	12	*C. felis*	9	3	_/	_/
Ouacif	36°31′25″ N, 4°12′20″ E	Dog	8	*R. sanguineus s.l*	3	5	_/	_/
			24	*R. turanicus*	12	12	/_	_/
		Dog	8	*R. sanguineus s.l*	3	5	3	5 (2♀, 3♂)
			24	*R. turanicus*	12	12	16	8 (5♀, 3♂)
		Rabbit	9	*C. felis*	8	1	_/	_/
Tizi Rached	36°33′45″ N, 2°32′00″ E	Dog	5	*R. sanguineus s.l*	0	5	0	5♂
			59	*R. turanicus*	37	22	49	10 (7♀, 3♂)
		Dog	9	*R. sanguineus s.l*	1	8	/_	/_
			25	*R. turanicus*	8	17	_/	/_
		Cat	3	*R. sanguineus s.l*	0	3	3	0
			32	*R. turanicus*	16	16	12	20 (12♀, 8♂)
		Dog	3	*C. felis*	3	0	0	3♀
Bouzgeune	36°37′00″ N, 4°28′47″ E	Dog	7	*R. sanguineus s.l*	6	1	7	0
			10	*R. turanicus*	3	7	10	0
		Dog	24	*R. sanguineus s.l*	11	13	/_	_/
			92	*R. turanicus*	63	29	_/	_/
		Cat	6	*R. sanguineus s.l*	6	0	6	0
			2	*R. turanicus*	1	1	2	0
		Cat	3	*R. sanguineus s.l*	3	0	_/	_/
		Goat	2	*R. sanguineus s.l*	2	0	_/	/_
			6	*R. turanicus*	6	0	_/	/_
		Goat	13	*C. felis*	9	4	13	0
		Sheep	12	*C. felis*	11	1	11	1♀
		Sheep	6	*C. felis*	4	2	_/	_/
		Cat	10	*C. felis*	10	0	7	3♀
Mekla	36° 41′ 16″ N, 4° 16′ 05″ E	Dog	8	*R. sanguineus s.l*	0	8	/	_/
			36	*R. turanicus*	17	19	/_	_/
		Rabbit	11	*C. felis*	11	0	11	0
		Rabbit	1	*C. felis*	1	0	_/	_/
		Sheep	9	*C. felis*	8	1	_/	_/
M’sila	35°42′07″ N, 4°32′48″ E	Dog	3	*R. sanguineus s.l*	0	3	0	3♂
			17	*R. turanicus*	11	6	0	17 (11♀, 6♂)
Messloula	35°24′19″ N, 8°06′59″ E	Dog	2	*R. sanguineus s.l*	1	1	1	1♂
			1	*R. turanicus*	1	0	0	1♂
		Total ectoparasites	599		359	240	/	/
			505 ticks/94 fleas		279 ticks/80 fleas	226 ticks/14 fleas	/	/
		Total ectoparasites tested for *Leishmania* DNA	247		155♀	92♂	157 (101♀, 56♂)	90 (53♀, 37♂)

**Table 2:** Geographical distribution of ectoparasites harboring *Leishmania* DNA in Algeria.

Ectoparasites/Hosts		*R. sanguineus s.l*		*R. turanicus*		*C. felis*		Total	
		N° Positive	(%)	N° Positive	(%)	N° Positive	(%)	N° Positive	(%)
**Tizi-Ouzou**	Draâ Ben Khedda	5♀/7	42.85	6 (4♂, 2♀)/10	60.00	2♀/2	100.00	13/19	68.42
	Ouacif	5 (3♂, 2♀)/8	62.5	8 (3♂, 5♀)/24	33.33	-	-	13/32	40.62
	Tizi Rached	5♂/8	62.5	30 (11♂, 19♀)/91	32.96	3♀/3	100.00	38/102	37.25
	Bouzgeune	0/13	0.00	0/12	0.00	4♀/35	11.42	4/60	6.66
	Mekla	-	-	-	-	0/11	0.00	0/11	0.00
	**Total**	**15/36**	**41**	**44/137**	**32.11**	**9/51**	**17.64**	**68/224**	**30.35**
**M’sila**		3♂/3	100.00	17(6♂, 11♀)/17	100.00	-	-	20/20	100.00
**Tébessa**		1♂/2	50.00	1♂/1	100.00	-	-	2/3	66.66
**Total**		**19/41**	**46.34**	**62/155**	**40.00**	**9/51**	**17.64**	**90/247**	**36.43**

**Table 3:** Host-specific distribution of ectoparasites harboring *Leishmania* DNA.

Ectoparasite/Hosts	*R. sanguineus*		*R. turanicus*		*C. felis*		Total	
	N° Positive	(%)	N° Positive	(%)	N° Positive	(%)	N° Positive	(%)
**Dogs**	19 (12♂, 7♀)/30	63.33	40 (17♂, 23♀)/115	34.78	3♀/3	100	(62/148)	41.89
**Cats**	0/9	0.00	20 (8♂, 12♀)/34	58.82	3♀/10	30	(23/53)	43.39
**Sheeps**	0/2	0.00	2 (2♀)/6	33.33	3♀/14	21.42	(5/22)	22.72
**Goats**	-	-	-		0/13	0.00	(0/13)	0.00
**Rabbits**	-	-	-		0/11	0.00	(0/11)	0.00
**Total**	**19/41**	**46.34%**	**62/155**	**40.00%**	**9/51**	**17.64%**	**90/247**	**36.43%**

**Table 4:** Association between ectoparasite sex and *Leishmania* DNA detection (Chi-Square Test).

Ectoparasites	Positive		Negative		*p* Valus > 0.05
	♀	♂	♀	♂	
***R. sanguineus s.l***	7	12	15	7	0.06
***R. turanicus***	37	25	49	44	0.32
***C. felis***	9	0	37	5	0.999
**Total**	**53**	**37**	**101**	**56**	**247**

**Table 5:** Predictors of *Leishmania* DNA positivity in ectoparasites: logistic regression results.

Predictor	Coefficient	Std. Error	z-Score	*p*-Value	95% CI (Lower)	95% CI (Upper)
Intercept	−0.213	0.509	−0.42	0.675	−1.210	0.784
*R. sanguineus s.l*	+0.147	0.593	0.25	0.804	−1.015	1.310
*R. turanicus*	−0.122	0.533	−0.23	0.819	−1.166	0.922
Dog	−0.050	0.336	−0.15	0.882	−0.708	0.608
Sheep	−0.992	0.627	−1.58	0.113	−2.221	0.236

Reference categories: Ectoparasite = *C. felis*, Host = Cat.

## Data Availability

All data generated or analyzed during this study are included in this published article.

## Abbreviations

The following abbreviations are used in this manuscript:

AAAES	Algerian Association for Animal Experimentation Sciences
ANOVA	Analysis of variance
bp	base pair(s)
CanL	Canine leishmaniasis
CCL	Chronic cutaneous leishmaniasis
CI	Confidence interval
CL	Cutaneous leishmaniasis
CTAB	Cetyltrimethylammonium bromide
DNA	Deoxyribonucleic acid
GPS	Global Positioning System
ITS1	Internal transcribed spacer 1
KOH	Potassium hydroxide
(*L.*)	*Leishmania* (genus abbreviation)
OR	Odds ratio
PCR	Polymerase chain reaction
qPCR	Quantitative polymerase chain reaction
RFLP	Restriction fragment length polymorphism
*s.l.*	*sensu lato* (in the broad sense)
*s.s.*	*sensu stricto* (in the strict sense)
spp.	multiple species within a genus
SCL	Sporadic cutaneous leishmaniasis
TE (buffer)	Tris–EDTA buffer
UV	Ultraviolet
VL	Visceral leishmaniasis
WHO	World Health Organization
ZCL	Zoonotic cutaneous leishmaniasis
